# Factors Influencing Acromial and Scapular Spine Strain after Reverse Total Shoulder Arthroplasty: A Systematic Review of Biomechanical Studies

**DOI:** 10.3390/jcm11020361

**Published:** 2022-01-12

**Authors:** Alexander Paszicsnyek, Olivia Jo, Harshi Sandeepa Rupasinghe, David C. Ackland, Thomas Treseder, Christopher Pullen, Greg Hoy, Eugene T. Ek, Lukas Ernstbrunner

**Affiliations:** 1 Department of Orthopaedics and Traumatology, Paracelsus Medical University, Strubergasse 21, 5020 Salzburg, Austria; ap@ortho-unfall.at; 2 Department of Orthopaedic Surgery, Royal Melbourne Hospital, 300 Grattan Street, Parkville, Melbourne, VIC 3050, Australia; jo.olivia1310@gmail.com (O.J.); tomtreseder@me.com (T.T.); cmpullen@bigpond.com (C.P.); 3 Department of Biomedical Engineering, University of Melbourne, Parkville, Melbourne, VIC 3010, Australia; rupasingheh@student.unimelb.edu.au (H.S.R.); dackland@unimelb.edu.au (D.C.A.); 4 Melbourne Orthopaedic Group, Windsor, VIC 3181, Australia; gahoy@mog.com.au (G.H.); eugene_ek@me.com (E.T.E.)

**Keywords:** reverse total shoulder arthroplasty, acromion fracture, scapular spine fracture, design parameters, biomechanics

## Abstract

Background: Acromial and scapular spine fractures after reverse total shoulder arthroplasty (RTSA) can be devastating complications leading to substantial functional impairments. The purpose of this study was to review factors associated with increased acromial and scapular spine strain after RTSA from a biomechanical standpoint. Methods: A systematic review of the literature was conducted based on PRISMA guidelines. PubMed, Embase, OVID Medline, and CENTRAL databases were searched and strict inclusion and exclusion criteria were applied. Each article was assessed using the modified Downs and Black checklist to appraise the quality of included studies. Study selection, extraction of data, and assessment of methodological quality were carried out independently by two of the authors. Only biomechanical studies were considered. Results: Six biomechanical studies evaluated factors associated with increased acromial and scapular spine strain and stress. Significant increases in acromial and scapular spine strain were found with increasing lateralization of the glenosphere in four of the included studies. In two studies, glenosphere inferiorization consistently reduced acromial strain. The results concerning humeral lateralization were variable between four studies. Humeral component neck-shaft angle had no significant effect on acromial strain as analysed in one study. One study showed that scapular spine strain was significantly increased with a more posteriorly oriented acromion (55° vs. 43°; *p* < 0.001). Another study showed that the transection of the coracoacromial ligament increased scapular spine strain in all abduction angles (*p* < 0.05). Conclusions: Glenoid lateralization was consistently associated with increased acromial and scapular spine strain, whereas inferiorization of the glenosphere reduced strain in the biomechanical studies analysed in this systematic review. Humeral-sided lateralization may increase or decrease acromial or scapular spine strain. Independent of different design parameters, the transection of the coracoacromial ligament resulted in significantly increased strains and scapular spine strains were also increased when the acromion was more posteriorly oriented. The results found in this systematic review of biomechanical in-silico and in-vitro studies may help in the surgical planning of RTSA to mitigate complications associated with acromion and scapular spine fracture.

## 1. Introduction

The indications for reverse total shoulder arthroplasty (RTSA) are broad and include irreparable rotator cuff tear or arthropathy [1,2,3,4,5,6], complex proximal humerus fractures in elderly patients [7,8,9,10], and revision arthroplasty [11]. RTSA is designed to medialize the glenohumeral joint center of rotation through offset lateralisation and inferiorization of the humerus, thereby increasing the deltoid moment arm. This in turn decreases the required deltoid force to combat gravity during abduction [12]. However, the inherent change in upper limb biomechanics, whilst offering advantages such as increased abduction and stability [13], results in increased stresses on the acromion and scapular spine [14]. Acromial and/or scapular spine fractures are relatively common complications of RTSA, occurring in up to 10% of patients [15,16,17,18,19,20,21,22]. These fractures have been associated with a substantial decline in outcomes with a reduced range of motion [22,23] and increased pain [24]. The management of these fractures, particularly Levy zone II and III fractures [15,20,25,26] is challenging [20,24,27] and associated with high rates of malunion or non-union [16,17,20,22]. As a result, there has been increasing interest in preventing these fractures from occurring in the first place [16,17,20,22].

Patient factors such as female gender, osteoporosis, or acromial anatomy have been identified as risk factors for acromial and scapular spine fractures after RTSA [28,29,30]. Implant factors, such as increased lateralization of the glenoid component, were proposed to play a significant role in increasing stress on the acromion [31,32]. It has also been suggested that combined medialization and proximalisation of the glenoid component, and hence the joint center of rotation, is associated with acromial fractures [33]. While lowering of the humerus is thought to increase acromial stress through excessive tensioning of the deltoid [27,34], humeral lateralization may have a protective effect against fracture [33]. The exact biomechanics of acromial and scapular spine strain patterns remain poorly understood. 

The purpose of this systematic review was therefore to analyse the biomechanical impact of different RTSA design features on acromion and scapular spine strain after RTSA and to identify potential implant- and anatomy-related risk factors for acromial and scapular spine fractures in RTSA. 

## 2. Material and Methods

### 2.1. Search Strategy

The systematic review followed the Preferred Reporting Items of Systematic Reviews and Meta-analysis (PRISMA) guidelines (Supplementary Material, File S1) [35], and was registered in the International Prospective Register for Systematic Reviews and Meta-analysis (PROSPERO): CRD42021297115 (Supplementary Material, File S2). A systematic search was conducted of PubMed, Embase, OVID Medline, and CENTRAL (Cochrane Central Register of Controlled trials) databases. The following keywords were used for the search: “reverse shoulder arthroplasty”, “reverse total shoulder prosthesis”, “reverse shoulder prosthesis” were combined with “acromial fracture”, “acromial strain”, “acromial pathology”, “acromial stress”, as well as “scapular spine fracture”, “scapular spine strain”, “scapular spine pathology”, and “scapular spine stress” (Supplementary Material, File S3). 

### 2.2. Selection Process

Two authors (A.P., O.J.) independently screened titles, abstracts, and full texts using the predefined inclusion and exclusion criteria. In cases of discrepancy, the senior author (L.E.) was consulted until a final consensus was reached. Biomechanical studies reporting acromial and scapular spine strains or stress after RTSA were chosen based on the following inclusion criteria:

(1) Biomechanical in-vitro or in-silico studies;
(2) Studies reporting on acromion or scapular spine fracture, strain, and stress;
(3) Studies in the German or English language;
(4) Studies released between January 2015 and May 2021.

We excluded editorial comments, review articles, conference proceedings, outcome studies, and studies that were not in the English or German language. 

### 2.3. Data Interpretation

**Figure 1:**
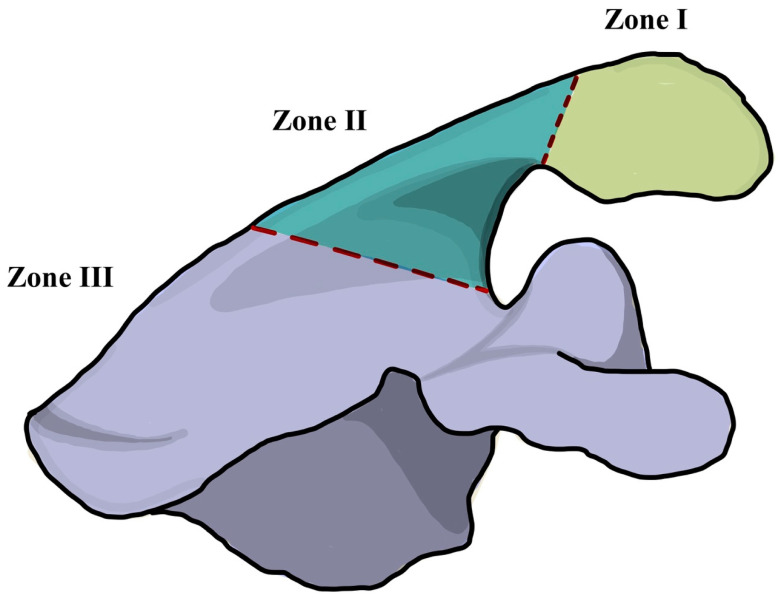
Illustration of the Levy classification for acromial stress fractures after reverse total shoulder arthroplasty [20]. Zone I involves a fracture through the midpart of the acromion caused by the anterior and middle deltoid origin. Zone II was defined as a fracture caused by the entire middle deltoid segment and portion of posterior deltoid origin. Zone III fractures involve the entire middle and posterior deltoid origin.

Basic study designs, number of specimen, type of study, and measuring locations were collected, as well as all aspects of implant design and acromial and scapula anatomy that may influence acromial and/or scapular strain after RTSA. Factors defined for this analysis included glenoid-sided and humeral-sided offset, deltoid lengthening, glenosphere inferiorization, neck-shaft angle (NSA), acromial morphology, and coracoacromial ligament integrity. A total of 25 mm of deltoid lengthening was used as a threshold value as it was associated with a decline in shoulder function in a previous study [36]. Stress and strain values obtained by the biomechanical studies were recorded. Stress is defined as the ratio of the force to the cross-sectional area and strain is defined as the relative deformation of a material when a given force is applied [37]. Information on the stress by location was also extracted based on the Levy classification for acromial stress fractures [20]. Type I involves a fracture through the midpart of the acromion caused by the anterior and middle deltoid origin. Type II was defined as a fracture caused by the entire middle deltoid segment and portion of posterior deltoid origin. Type III fractures involve the entire middle and posterior deltoid origin (Figure 1).

### 2.4. Study Quality Assessment

A risk of bias assessment was conducted using a modified Downs and Black checklist [38]. This checklist is a quality assessment tool used for systematic reviews of biomechanical studies [39]. With a maximum possible score of 12, the methodological quality of the included studies was classified into high (>9 points), moderate (6–8 points), and low (<5 points). Two reviewers (A.P. and O.J.) assessed studies independently. In case of inter-rater disagreement, the senior author (L.E.) was consulted until a final consensus was reached. 

## 3. Results

### 3.1. Search Results

**Figure 2:**
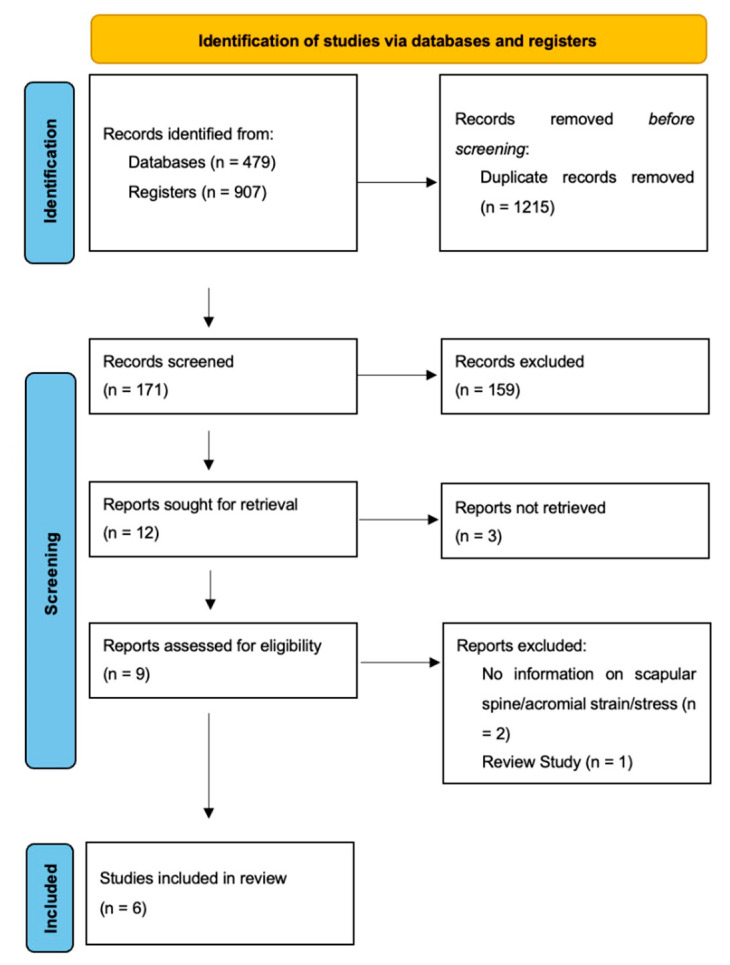
PRISMA flow diagram of the systematic search.

Using combinations of the predefined search terms, a total of 1386 studies were identified. A total of 171 studies remained after the removal of duplicates and screening of titles. After abstract screening, 12 studies underwent full-text evaluation. Three studies were excluded as full-text articles were not available [40,41,42]. Two studies did not report on scapular strain or stress [43,44], and one study was a systematic review [45]. Six studies [14,29,30,46,47,48] met the inclusion and exclusion criteria and were included in this study (Figure 2).

### 3.2. Study Quality Assessment

In this review, 5 studies [14,29,30,46,47] were of high methodological quality and one study [48] was of moderate quality due to the limited number of specimens included (*n* = 1) and lack of appropriate statistical analysis. 

### 3.3. Study Characteristics

**Figure 3:**
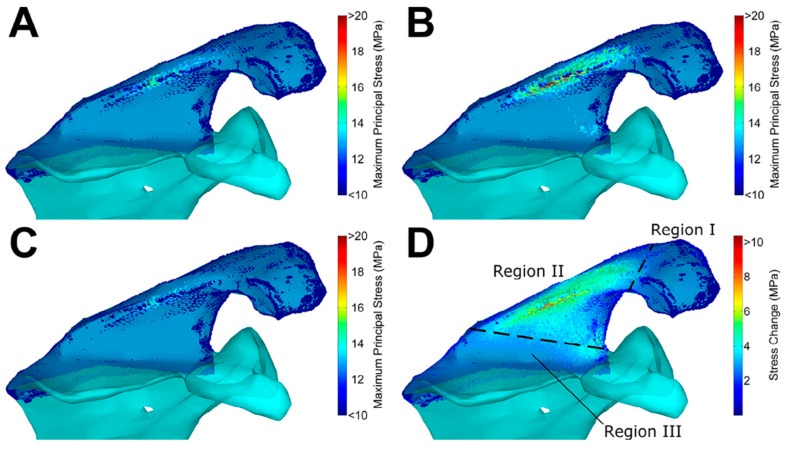
Finite element analysis on the influence of implant positioning in reverse total shoulder arthroplasty on acromial and scapular spine stresses by Wong et al. [47]. Reprinted with permission from ref. [47]. Copyright 2016 Elsevier. (**A**) Baseline implant configuration, (**B**) maximum stress configuration, (**C**) minimum stress configuration, and (**D**) stress increase from minimum to maximum stress configuration. Regions are as defined by Levy et al. [20].

Three of the included studies were in-silico studies consisting of computational analyses, with all of them using finite element (FE) modelling [14,48,49] (Figure 3). 

**Figure 4:**
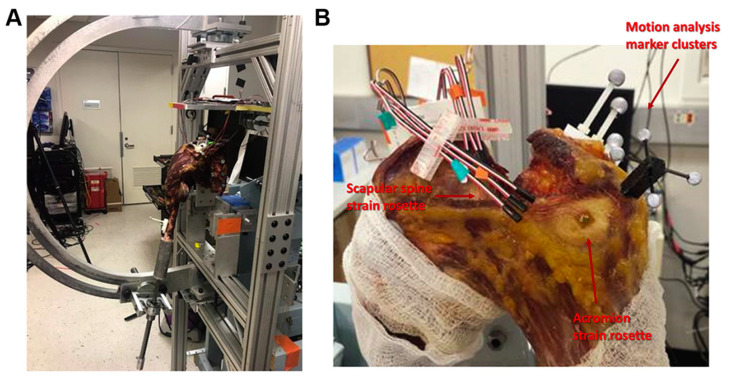
Biomechanical set up of the study by Shah et al. [29]. Reprinted with permission from ref. [29]. Copyright 2020 Elsevier. Biomechanical testing apparatus. (**A**) The humerus was mounted to a 6-degree-of-freedom shoulder simulator that can simulate different levels of abduction. (**B**) Strain rosettes (Vishay Measurements Group), 3 strain gauges overlapping and patterned in a 90 angle, were rigidly glued on to the surface of the acromial body and the scapular spine to represent the locations of Levy et al. [20] type II and type III fractures, respectively.

The other 3 in-vitro studies used cadaveric models in combination with a shoulder simulator in different shoulder positions [29,30,46] (Figure 4). 

A total of 46 cadavers were used in the included studies. Five studies [14,29,30,46,48] measured strain/stress according to the Levy zones [20] (Table 1). 

### 3.4. Glenoid Lateralization

**Figure 5:**
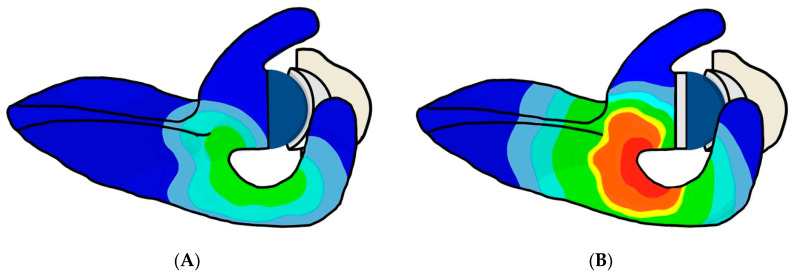
Illustration of the influence of glenoid component lateralization on acromial and scapular spine strain. Acromial and scapular spine strain are increased when comparing a (**A**) non-lateralized glenoid component with a (**B**) lateralized glenoid component.

Four studies of high to moderate qualities reported on the influence of glenoid component lateralization on acromial and scapular spine strain [14,29,47,48], and showed a consistent increase in acromial and scapular spine stress and strain with glenoid lateralization (Figure 5). 

Lockhart et al. [14] showed in their computational study that the lateralization of the glenoid component by 10 mm increased the maximum stress on the acromion during abduction by 9.0 MPa compared to the unlateralized condition (16%, *p* < 0.001). During forward elevation, maximum acromial stress increased by 4.0 MPa (11%, *p* = 0.009) at 5-mm lateralization and by 8.0 MPa (21%, *p* < 0.001) at 10-mm lateralization. In an in-silico study by Wong et al. [47], the authors reported a significant increase of 4.1 MPa (17%, *p* = 0.003) in acromial stress during abduction with a glenosphere lateralized by 10 mm compared to the 0-mm lateralization condition. Furthermore, Zeng et al. [48] showed that 12 mm of glenosphere lateralization increased deltoid force by 16% and peak acromial stress by 11.6 MPa compared to 0-mm lateralization in their computational analyses. Shah et al. [29] demonstrated that in all humeral onlay conditions, lateralization of the glenosphere increased acromial and scapular spine strains in their cadaveric study. A 6-mm increase in glenosphere lateralization with a +10-mm lateralized humeral onlay tray resulted in a significant increase in acromial strain when compared to 0 mm of lateralization (905 µε vs. 962 µε; 6%, *p* = 0.029). Similarly, with a +13-mm lateralized humeral onlay, acromial strain increased from 962 µε to 1112 µε (14%, *p* = 0.048) when the glenosphere was lateralized by 6 mm. 

### 3.5. Glenoid Inferiorization

The effect of inferiorization of the glenosphere on acromial stress was assessed in 2 in-silico studies of high quality [14,47]. Lockhart et al. [14] found a significant reduction in peak acromion stress during abduction with 2.5-mm glenosphere inferiorization by 1.8 MPa (3%, *p* = 0.001), while 5-mm inferiorization resulted in a greater decrease by 3 Mpa (5%, *p* = 0.002) in acromial stress when compared with 0 mm of inferiorization. Significant reduction in acromial stress was also observed in the sagittal plane by 2 MPa (5%, *p* = 0.041) for 5 mm of inferiorization. Wong et al. [47] found a reduction of acromial stress during abduction by 0.4 MPa (2%, *p* = 0.008) with 2.5 mm of inferiorization, while a decrease by 0.7 MPa (3%, *p* = 0.024) was noted with 5 mm of inferiorization. 

### 3.6. Humeral Lateralization

Four studies of high quality reported the effect of lateralization of the humerus on acromial stress during glenohumeral abduction [14,29,46,47]. In the cadaveric study by Shah et al. [29], incremental increase of humeral onlay (+3, +5, +8, +10, +13 mm) resulted in significantly increased maximum strains on the acromion (i.e., 348 µε vs. 427 µε vs. 609 µε vs. 696 µε vs. 962 µε, respectively; *p* < 0.05). Similarly, Wong et al. [47] showed that during abduction, lateralization of the humerus increased acromial stresses by 1.7% (0.8 MPa; *p* = 0.051), whereas medialization by 5 mm decreased stresses by 1.4% (0.4 MPa; *p* = 0.038). On the other hand, Kerrigan et al. [46] reported a decrease of strain on the acromion of 34% with increased lateralization in their in-vitro study (−5 mm medialization vs. 15 mm lateralization; *p* = 0.042). Lastly, Lockhart et al. [14] reported that humeral offset had no significant effect on acromial strain regardless of the direction of offset (medial or lateral) nor the plane of elevation (abduction or forward elevation). 

### 3.7. Deltoid Lengthening

The influence of deltoid lengthening was evaluated in the in-vitro study by Shah et al. [29]. They showed that deltoid length was increased by 16 mm with the standard onlay humeral insert and standard glenosphere compared to that in the native shoulder. Deltoid length significantly increased to 29 mm with the +13 mm humeral onlay insert (*p* < 0.01). The peak deltoid lengthening of 31 mm was observed with a +6-mm lateralized glenosphere combined with a +13-mm humeral insert. This maximal deltoid lengthening was associated with an acromial strain of 1112 µε, which was an increase of 83% in strain compared to that at 25 mm of deltoid lengthening (*p* = 0.012). At all other configuration of implants resulting in deltoid lengthening beyond 25 mm, a 79-µε incremental increase in acromial stress per mm lengthening was observed.

### 3.8. Neck-Shaft Angle 

The influence of NSA was only reported in one study [46], and did not result in a statistically significant effect (*p* > 0.05) on acromial or scapular stress when the NSA was changed between 135°, 145°, and 155°. 

### 3.9. Acromial Morphology

Shah et al. reported on the influence of acromial morphology on scapular spine and acromial strains [29]. Of the 10 cadaveric shoulders tested, five shoulders (group A) had higher strain on the scapular spine than on the acromion (1445 µε vs. 862 µε; *p* = 0.02) at maximal deltoid lengthening. Meanwhile, the remaining five shoulders (group B) showed higher strain on the acromion than on the scapular spine (1203 µε vs. 603 µε; *p* = 0.003) at the same degree of deltoid lengthening. They found that group A, with higher scapular spine strain than acromial strain, had a larger mean SSA (i.e., flatter scapular spine), compared to group B, with higher acromial strain than scapular strain (55° vs. 43°; *p* < 0.001). Furthermore, group A (higher strain on scapular spine) was found to have a more posteriorly oriented acromion than group B (higher strain on acromion), which had an acromion that was placed more anteriorly (−5.3 mm vs. 6.7 mm, *p* < 0.001). 

### 3.10. Coracoacromial Ligament

One in-vitro study [30] reported the influence of the coracoacromial ligament on acromial and scapular spine strain after RTSA in a cadaveric model. Following coracoacromial ligament transection, scapular spine strain at all abduction angles was significantly increased compared with that in the intact condition (*p* < 0.05). The peak scapular spine strain increased by 19.7% following coracoacromial transection (1.216 µε; *p* = 0.011). 

## 4. Discussion

The effects of humeral and glenoid lateralization of a non-Grammont design are to bring the lesser and greater tuberosities to a more anatomic position than with the traditional medialized design and to facilitate two important aspects [50]: (1) Increased resting tension of the remaining rotator cuff and deltoid, thus increasing compressive forces on the joint and thus increasing joint stability [31,32,51,52]; and (2) increased wrapping of the deltoid, thus increasing the horizontal stability through compressive force [53,54]. Besides these improved biomechanics of modern RTSA, changing the glenoid offset can have adverse effects, such as reducing the moment arm of the deltoid muscle as well as creating large bending moments at the base-plate fixation, which may create an environment where fixation failure is more likely [55].

One of the main findings of this study was that glenoid lateralization significantly and consistently increased acromial and scapular spine stress and strain. As lateralization of the center of rotation reduces the moment arm of the deltoid muscle, increased deltoid forces for abduction are necessary [31,32]. This leads to an increased acromion and scapular spine strain [13,32]. Glenoid lateralization can improve clinical and radiographic outcomes, but it can also be associated with increased acromial and scapular spine strain, and should therefore be considered as a risk factor of acromion and scapular spine fractures, following RTSA. 

Inferiorization of the glenosphere uniformly reduced acromial stress across the included studies. This is likely due to the lengthening of the deltoid and a shift in the center of rotation towards a larger deltoid moment arm for abduction. An increased moment arm reduces deltoid forces, thereby reducing forces directly applied to the acromion [14]. In a cadaveric model including 8 shoulders, RTSA with a 2.5-mm glenosphere inferiorization compared to a 4-mm lateralized glenosphere resulted in a reduction in deltoid force to abduct the arm [56]. Therefore, glenosphere inferiorization in combination with glenosphere lateralization (if desired) may neutralize acromion and scapular spine strain, although glenoid lateralization seems to have a larger effect on acromial stress than inferiorization [49]. 

Lateralization of the humerus has shown variable effects on acromial and/or scapular spine strains. Wong et al. [49] showed in a computational study that, during abduction, lateralization of the humerus increased acromial stress, whereas medialization of the humerus results in significantly decreased acromial stress. The authors believe that this was due to the decreased passive stretch and tensioning of the deltoid with humeral medialization. Shah et al. [29] incrementally increased humeral lateralization and found that onlay system lateralization results in significant deltoid lengthening. This results in a subsequent increase in passive tension in the deltoid and the overall force acting on the acromion and scapular spine, which therefore, increases strain. On the other hand, Kerrigan et al. [46] reported that humeral lateralization caused significant decreases in scapular spine strain during abduction. They hypothesized that increasing humeral lateralization results in a larger moment arm for the deltoid in abduction, which decreased the deltoid force necessary to abduct, which reduces acromial and scapular strain. Accordingly, Giles et al. [31] further demonstrated in a cadaveric model that humeral lateralization decreased the deltoid force required for active abduction due to the increased muscle moment arm. Based on these results, humeral lateralization results in two effects that interplay: (1) It increases the passive tension of the deltoid, resulting in increased force acting on the acromion and scapular spine and (2) it increases the muscle moment arm and therefore decreases the active force necessary for active abduction. The more dominant effect may depend on a number of factors, including implant design. Furthermore, because all the involved studies have used onlay humeral trays, there may be some differences in effects on an inlay model. 

The effect of changing NSA on the shoulder range of motion and scapular notching has been studied extensively [57,58,59,60,61,62]. Implants with more anatomical or varus humeral angles produce increased adduction and external rotation [60], and are less prone to scapular impingement [57]. The impact of the humeral neck-shaft angle on scapular spine strain is less clear and was assessed in one of the included studies [46]. It was found that varying NSA did not influence scapular spine strain in any of the four planes of elevation. This may be because humeral inclination has little [60] or no effect [46] on humeral offset. Thus, more varus humeral component NSA may offer the advantage of reduced scapular notching whilst having minimal impact on scapular spine strain.

Acromial morphology has been shown to influence the distribution of strain on the acromion as well as the scapular spine. Shah. et al. [29] described the influence of acromial and scapular spine orientation in the parasagittal plane on strain patterns. A flatter scapular spine in combination with a more posteriorly oriented acromion resulted in a significantly higher strain burden on the scapular spine in comparison to the acromion. Conversely, a more vertically oriented scapular spine in combination with a more anteriorly oriented acromion resulted in a significantly higher strain on the acromion than on the scapular spine. The exact mechanism by which anatomical changes in the scapula influence acromial and scapular spine strain is unclear. Furthermore, another (unknown) factor to consider in order to predict strain tendencies during preoperative planning of RTSA is the influence of thoracic kyphosis [63].

The coracoacromial ligament plays a role in transmitting forces acting on the acromion to the coracoid process and vice versa [64,65]. Taylor et al. [30] showed in a cadaveric study that transecting the coracoacromial ligament results in significantly increased scapular spine strain at all abduction angles. The authors suggest that the coracoacromial ligament alters strain patterns along the acromion and scapular spine. This is a result of the counterbalance role of the coracoacromial ligament. The deltoid creates a cantilever as a result of the shape of the acromion, resulting in the bending of the acromion and therefore raising the strain affecting the scapular spine. This is normally counteracted by the coracoacromial ligament and therefore transection results in an alteration of strain patterns along the acromion and scapular spine [30]. Clinically, preserving the coracoacromial ligament was associated with a significant reduction of acromial stress reactions and occult fractures following RTSA in a study involving 265 patients [66]. Therefore, maintaining the coracoacromial ligament integrity may be a modifiable risk factor for acromial fractures following RTSA. 

The location of the acromion or scapular spine fracture not only influences patient outcome, but also plays an important role in the choice of treatment [17,18,22]. Based on the Levy classification [20], type II fractures are most common (50%), followed by type III (38%), and type I fractures (12%). The four biomechanical studies that analysed the influence of the Levy zones on acromial and scapular spine strain [14,46,47,48] confirmed this finding by observing the highest stress and strain values in zone II and III, respectively. In the study by Zeng et al. [48], strain was highest in zone II. Wong et al. [47] also located the highest stress in Levy zone II, followed by zone III and zone I. Similarly, Kerrigan et al. [46] measured highest strain in zone II. In a study by Lockhart et al. [14], the stress in zone II was the greatest regardless of implant configuration, loads, and plane of elevation, followed by zone III and zone I. 

The Levy zones also play a relevant role in the treatment of acromial and scapular spine fractures. Type I and some type II fractures can be treated non-operatively [24], with a moderate union rate of about 50% and an acceptable functional outcome [17,22]. Type III fractures are challenging to treat as the broad deltoid muscle insertion and poor fragment bone stock compromise stable fixation [15,20,25,26]. Although open reduction and internal fixation is the preferred treatment method, it is associated with a high non-union rate [16,22,67,68]. Similarly, non-operative management with an abduction splint is also associated with a high non-union rate and does not reveal superior results over surgical fixation [16,22]. The resulting tilt of the most lateral scapular fragment leads to impingement, reduced range of motion, and ongoing pain [23]. Therefore, acromial and scapular spine fractures after RTSA are not only a common problem but also hard to treat [15,17,24,69,70].

There are several limitations to this study. Firstly, PROSPERO registration was conducted after completion of this systematic review. However, the study protocol was strictly followed and has not been changed during the conduction of this study nor before submission to PROSPERO. Secondly, the included studies reported on varying implant factors that could affect stress on the acromion. In all, there was limited data to allow meta-analysis. Nonetheless, a comprehensive description of data and comparison were possible to derive a meaningful discussion. Thirdly, the studies involved were either computational analyses or cadaveric studies. These have inherent limitations in replicating results in in-vivo biomechanics and physiology. However, these studies were conducted with consistent design and testing protocols in the exclusion of other potential interfering variables, such as the rotator cuff. This provides accurate results on true strain/stress response at the acromion and scapular spine resulting from altered deltoid forces. Fourthly, the base implant models were varied with differing NSA between studies. The impact of this in interpreting and comparing results is uncertain. Finally, acromial and scapular spine fractures are the result of bony stress of a certain cross-sectional area exceeding the bony strength in this area. Although this study discussed factors influencing bony stress, it did not consider bony acromial and scapular spine strength. Risk factors such as osteoporosis, cuff tear arthropathy, inflammatory arthritis, or older age are known to be associated with acromial and scapular spine fractures following RTSA [71]. When planning RTSA, both factors altering bony stress as well as factors affecting bone strength need to be considered and this combination of factors should be studied in future. 

## 5. Conclusions

Glenoid lateralization was consistently associated with increased acromial and scapular spine strain, whereas inferiorization of the glenosphere reduced strain in the biomechanical studies analysed in this systematic review. Humeral-sided lateralization may increase or decrease acromial or scapular spine strain. Independent of different design parameters, transection of the coracoacromial ligament resulted in significantly increased strains, and scapular spine strains were also increased when the acromion was more posteriorly oriented. The results found in this systematic review of biomechanical in-silico and in-vitro studies may help in surgical planning of RTSA to mitigate complications associated with acromion and scapular spine fractures. 

## Figures and Tables

**Table 1:** Characteristics of the included studies.

Study	Specimen Type, Number, Age (Range)	Study Type	Implant Used	NSA (°)	Humeral Offset (mm) *	Glenosphere Offset (mm) *	Outcome(s) Assessed	Scapular Strain/Stress Location ^†^
Kerrigan (2021) [46], Canada, DCOI	Cadaveric, 8, 73 (61–88)	In-vitro biomechanical study (quasi-static)	Custom modular 42 mm glenosphere Onlay humeral tray. No further specifications	135, 145, 155	−5.0, +5.0, +15.0	Lateralization: +5.0	Scapular strain during: A: Abduction (0–90°) in scapular plane and forward elevation (0–90°); B: Humeral lateralization; C: Varying neck shaft angles	Levy region I, II, and III
Lockhart (2020) [14], Canada	CT images of cadaveric shoulders, 10, 68 (49–87)	In-silico finite element modelling (quasi-static)	38 mm glenosphere Onlay humeral tray. No further specifications	155	+15.0, +20.0, +25.0	Inferiorization: 0, 2.5, 5.0 Lateralization: 0, +5.0, +10.0	Acromial stress during: A: Abduction (0°), scapular plane elevation (30°), forward elevation (60°) B: Loading (0, 2.5, 5 kg) C: Glenosphere lateralization D: Glenosphere inferiorization E: Humeral medialization and lateralization	Levy region I, II, and III
Shah (2020) [29], USA	Cadaveric, 10, 53.2 (37–63)	In-vitro biomechanical study (quasi-static)	Zimmer Biomet Comprehensive 36 mm glenosphere. Onlay humeral tray	147	+3.0, +5.0, +8.0, +10.0, +13.0	Lateralization: 0, +6.0	Acromial and scapular strain and deltoid lengthening: A: Based on anatomical orientation of acromion B: During glenosphere lateralization C: During humeral lateralization	Levy region II and III
Taylor (2020) [30], USA	Cadaveric, 8, 68 (56.9–79.1)	In-vitro biomechanical study (dynamic)	Zimmer Biomet Comprehensive 36 mm glenosphere. Onlay humeral tray	147	+3.0	No change in offset	Maximal principal strains on the acromion and scapular strain when: A: Coracoacromial ligament intact B: Coracoacromial ligament transacted	Levy region II and III
Wong (2016) [47], Canada, DCOI	Cadaveric, 10, 68 (49–87)	In-silico finite element modelling (dynamic)	Delta Xtend, Depuy Synthes 38 mm glenosphere. Onlay humeral tray	155	−5.0, 0, +5.0	Inferiorization:0, 2.5, 5.0 Lateralization: 0, +5.0, +10.0	Acromial stress during: A: Abduction (0–120°) B: Glenosphere inferiorization C: Glenosphere lateralization D: Humeral medialization and lateralization	
Zeng (2021) [48], USA	CT images of representative female subject, 1	In-silico finite element modelling (dynamic)	Zimmer Anatomical Reverse 36 mm glenosphere. Onlay humeral tray	-	-	Lateralization: 0, +6.0, +12.0	A: Maximal principal strain, stress and von Milses stress on scapula during glenosphere lateralization B: Deltoid muscle forces during glenosphere lateralization	Levy region I, II, and III

NSA, neck-shaft angle; DCOI, declared conflict of interest; * Positive values indicate lateralization and negative values indicate medialization of the component; ^†^ Acromial and scapular spine stress and strain regions were classified according to the Levy classification [20].

## Supplementary Materials

The following are available online at https://www.mdpi.com/article/10.3390/jcm11020361/s1, File S1: PRISMA_2020_checklist, File S2: PROSPERO public record, File S3: search strategy.
