# Supplementary material for: Factors Influencing Acromial and Scapular Spine Strain after Reverse Total Shoulder Arthroplasty: A Systematic Review of Biomechanical Studies
Source: J Clin Med. 2022 Jan 12;11(2):361. doi: 10.3390/jcm11020361 (PMC8778957; doi:10.3390/jcm11020361)
Supplement: Supplementary file 1 [file jcm-11-00361-s001.zip › jcm-1488498-supplementary/Supplemental material S2_PROSPERO public record.pdf]

Systematic review

Please select one of the options below to edit your record. Either option will create a new version of the record - the existing version will remain unchanged.

1. * Review title.

Give the title of the review in English

Factors Influencing Acromial and Scapular Spine Strain After Reverse Total Shoulder Arthroplasty: A Systematic Review of Biomechanical Studies

2. Original language title.

For reviews in languages other than English, give the title in the original language. This will be displayed with the English language title.

3. * Anticipated or actual start date.

Give the date the systematic review started or is expected to start.

08/12/2021

4. * Anticipated completion date.

Give the date by which the review is expected to be completed.

01/02/2022

5. * Stage of review at time of this submission. [1 change]

This field uses answers to initial screening questions. It cannot be edited until after registration.

Tick the boxes to show which review tasks have been started and which have been completed.

Update this field each time any amendments are made to a published record.

The review has not yet started: No

Review stage	Started	Completed
Preliminary searches	Yes	Yes
Piloting of the study selection process	Yes	Yes
Formal screening of search results against eligibility criteria	Yes	Yes
Data extraction	Yes	Yes

Risk of bias (quality) assessment	Yes	Yes
Data analysis	Yes	Yes

Provide any other relevant information about the stage of the review here.

Protocol was registered after completion of the review as the study protocol was strictly followed and not changed within the process of the study creation.

Protocol was registered after completion of the review as the study protocol was strictly followed and not changed within the process of the study creation.

6. * Named contact.

The named contact is the guarantor for the accuracy of the information in the register record. This may be any member of the review team.

Alexander Paszicsnyek

Email salutation (e.g. "Dr Smith" or "Joanne") for correspondence:
Mr Paszicsnyek

7. * Named contact email.

Give the electronic email address of the named contact.

ap@ortho-unfall.at

8. Named contact address

PLEASE NOTE this information will be published in the PROSPERO record so please do not enter private information, i.e. personal home address

Give the full institutional/organisational postal address for the named contact.

Paracelsus Medical University, Strubergasse 21, 5020 Salzburg, Austria

9. Named contact phone number.

Give the telephone number for the named contact, including international dialling code.

+4369914100135

10. * Organisational affiliation of the review.

Full title of the organisational affiliations for this review and website address if available. This field may be completed as 'None' if the review is not affiliated to any organisation.

Paracelsus Medical University, Strubergasse 21, 5020 Salzburg, Austria

Department of Orthopaedic Surgery, Royal Melbourne Hospital, Parkville, Victoria 3050, Australia

Melbourne Orthopaedic Group, Windsor, Victoria 3181, AustraliaDepartment of Biomedical Engineering, University of Melbourne, Parkville, Victoria 3010, Australia

Organisation web address:

11. * Review team members and their organisational affiliations.

Give the personal details and the organisational affiliations of each member of the review team. Affiliation refers to groups or organisations to which review team members belong.
NOTE: email and country now MUST be entered for each person, unless you are amending a published record.

Mr Alexander Paszicsnyek. Paracelsus Medical University
Dr Olivia Jo. Royal Melbourne Hospital
Dr Lukas Ernstbrunner. Royal Melbourne Hospital
Mr Harshi Sandeepa Rupasinghe. Melbourne Orthopedic Group
Professor David Ackland. Department of Biomechanical Engineering, University of Melbourne
Dr Thomas Treseder. Royal Melbourne Hospital
Dr Christopher Pullen. Royal Melbourne Hospital
Dr Greg Hoy. Department of Biomedical Engineering, University of Melbourne
Professor Eugene T Ek. Department of Biomedical Engineering, University of Melbourne

12. * Funding sources/sponsors.

Details of the individuals, organizations, groups, companies or other legal entities who have funded or sponsored the review.

None

Grant number(s)

State the funder, grant or award number and the date of award

13. * Conflicts of interest.

List actual or perceived conflicts of interest (financial or academic).

None

14. Collaborators.

Give the name and affiliation of any individuals or organisations who are working on the review but who are not listed as review team members. **NOTE: email and country must be completed for each person, unless you are amending a published record.**

15. * Review question.

State the review question(s) clearly and precisely. It may be appropriate to break very broad questions down into a series of related more specific questions. Questions may be framed or refined using PI(E)COS or similar where relevant.

Impact of alteration of components of RTSA on acromial/scapular spine strain

16. * Searches.

State the sources that will be searched (e.g. Medline). Give the search dates, and any restrictions (e.g. language or publication date). Do NOT enter the full search strategy (it may be provided as a link or attachment below.)

MEDLINE, Cochrane, PubMed, OVID

17. URL to search strategy.

Upload a file with your search strategy, or an example of a search strategy for a specific database, (including the keywords) in pdf or word format. In doing so you are consenting to the file being made publicly accessible.

Or provide a URL or link to the strategy. Do NOT provide links to your search **results**.

https://www.crd.york.ac.uk/PROSPEROFILES/297115_STRATEGY_20211208.pdf

Do not make this file publicly available until the review is complete

18. * Condition or domain being studied.

Give a short description of the disease, condition or healthcare domain being studied in your systematic review.

Acromial/Scapular spine strain/stress after RTSA

19. * Participants/population.

Specify the participants or populations being studied in the review. The preferred format includes details of both inclusion and exclusion criteria.

Cadaveric/finite element

20. * Intervention(s), exposure(s).

Give full and clear descriptions or definitions of the interventions or the exposures to be reviewed. The preferred format includes details of both inclusion and exclusion criteria.

RTSA and impact of alteration in components in biomechanical cadaveric and finite element studies on scapular spine/acromial stress/strain

21. * Comparator(s)/control.

Where relevant, give details of the alternatives against which the intervention/exposure will be compared (e.g. another intervention or a non-exposed control group). The preferred format includes details of both inclusion and exclusion criteria.

not relevant

22. * Types of study to be included.

Give details of the study designs (e.g. RCT) that are eligible for inclusion in the review. The preferred format includes both inclusion and exclusion criteria. If there are no restrictions on the types of study, this should be stated.

Biomechanical cadaveric and finite element studies

23. Context.

Give summary details of the setting or other relevant characteristics, which help define the inclusion or exclusion criteria.

24. * Main outcome(s).

Give the pre-specified main (most important) outcomes of the review, including details of how the outcome is defined and measured and when these measurement are made, if these are part of the review inclusion criteria.

Effect of rtsa component alteration on Strain/Stress on acromion and scapular spine
Glenosphere lateralization/inferiorization impact
Humeral medialization/lateralization impact

NSA Impact
CAL Ligament dissection impact

Measures of effect

25. * Additional outcome(s).

List the pre-specified additional outcomes of the review, with a similar level of detail to that required for main outcomes. Where there are no additional outcomes please state 'None' or 'Not applicable' as appropriate to the review

not applicable

Measures of effect

26. * Data extraction (selection and coding).

Describe how studies will be selected for inclusion. State what data will be extracted or obtained. State how this will be done and recorded.

Screening by two separate authors, senior author contacted if no consensus is found

27. * Risk of bias (quality) assessment.

State which characteristics of the studies will be assessed and/or any formal risk of bias/quality assessment tools that will be used.

Downs and Black (modified)

28. * Strategy for data synthesis.

Describe the methods you plan to use to synthesise data. This **must not be generic text** but should be **specific to your review** and describe how the proposed approach will be applied to your data.
If meta-analysis is planned, describe the models to be used, methods to explore statistical heterogeneity, and software package to be used.

extract and compare data of included studies and try to combine relevant topics, which may affect/have an impact on strain/stress at the Acromion and scapular spine

29. * Analysis of subgroups or subsets.

State any planned investigation of 'subgroups'. Be clear and specific about which type of study or participant will be included in each group or covariate investigated. State the planned analytic approach.

not relevant

30. * Type and method of review.

Select the type of review, review method and health area from the lists below.

Type of review

Cost effectiveness	No
Diagnostic	No
Epidemiologic	No

Individual patient data (IPD) meta-analysis	No
Intervention	Yes
Living systematic review	No
Meta-analysis	No
Methodology	No
Narrative synthesis	No
Network meta-analysis	No
Pre-clinical	No
Prevention	No
Prognostic	No
Prospective meta-analysis (PMA)	No
Review of reviews	No
Service delivery	No
Synthesis of qualitative studies	No
Systematic review	Yes
Other	No

Health area of the review

Alcohol/substance misuse/abuse	No
Blood and immune system	No
Cancer	No
Cardiovascular	No
Care of the elderly	No
Child health	No
Complementary therapies	No
COVID-19	No
Crime and justice	No

Dental	No
Digestive system	No
Ear, nose and throat	No
Education	No
Endocrine and metabolic disorders	No
Eye disorders	No
General interest	No
Genetics	No
Health inequalities/health equity	No
Infections and infestations	No
International development	No
Mental health and behavioural conditions	No
Musculoskeletal	Yes
Neurological	No
Nursing	No
Obstetrics and gynaecology	No
Oral health	No
Palliative care	No
Perioperative care	No
Physiotherapy	No
Pregnancy and childbirth	No
Public health (including social determinants of health)	No
Rehabilitation	No
Respiratory disorders	No
Service delivery	No
Skin disorders	No

Social care	No
Surgery	Yes
Tropical Medicine	No
Urological	No
Wounds, injuries and accidents	No
Violence and abuse	No

31. Language.

Select each language individually to add it to the list below, use the bin icon to remove any added in error.

English

There is not an English language summary

32. * Country.

Select the country in which the review is being carried out. For multi-national collaborations select all the countries involved.

Australia

Austria

33. Other registration details.

Name any other organisation where the systematic review title or protocol is registered (e.g. Campbell, or The Joanna Briggs Institute) together with any unique identification number assigned by them.
If extracted data will be stored and made available through a repository such as the Systematic Review Data Repository (SRDR), details and a link should be included here. If none, leave blank.

34. Reference and/or URL for published protocol.

If the protocol for this review is published provide details (authors, title and journal details, preferably in Vancouver format)

No I do not make this file publicly available until the review is complete

35. Dissemination plans.

Do you intend to publish the review on completion?

Yes

36. Keywords.

Give words or phrases that best describe the review. Separate keywords with a semicolon or new line. Keywords help PROSPERO users find your review (keywords do not appear in the public record but are included in searches). Be as specific and precise as possible. Avoid acronyms and abbreviations unless these are in wide use.

Reverse total shoulder arthroplasty; acromion fracture; scapular spine fracture; design parameters; biomechanics

37. Details of any existing review of the same topic by the same authors.

If you are registering an update of an existing review give details of the earlier versions and include a full bibliographic reference, if available.

38. * Current review status. [1 change]

Update review status when the review is completed and when it is published.
New registrations must be ongoing so this field is not editable for initial submission.

Review_Completed_not_published

39. Any additional information.

Provide any other information relevant to the registration of this review.

40. Details of final report/publication(s) or preprints if available.

Leave empty until publication details are available OR you have a link to a preprint (NOTE: this field is not editable for initial submission).
List authors, title and journal details preferably in Vancouver format.
