# Supplementary material for: Factors Influencing Acromial and Scapular Spine Strain after Reverse Total Shoulder Arthroplasty: A Systematic Review of Biomechanical Studies
Source: J Clin Med. 2022 Jan 12;11(2):361. doi: 10.3390/jcm11020361 (PMC8778957; doi:10.3390/jcm11020361)
Supplement: Supplementary file 1 [file jcm-11-00361-s001.zip › jcm-1488498-supplementary/Supplemental material S3_search strategy.pdf]

Medline
1. (RTSA* or reverse total shoulder arthroplasty* or reverse shoulder arthroplasty* or reverse total shoulder prosthesis* or reverse shoulder prosthesis*).mp.[mp=title, abstract, original title, name of substance word, subject heading word, floating sub-heading word, keyword heading word, organism supplementary concept word, protocol supplementary concept word, rare disease supplementary concept word, unique identifier, synonyms]<br>2. (Acromial fracture* or acromial pathology* or acromial strain* or acromial pathology*).mp.[mp=title, abstract, original title, name of substance word, subject heading word, floating sub-heading word, keyword heading word, organism supplementary concept word, protocol supplementary concept word, rare disease supplementary concept word, unique identifier, synonyms]<br>3. (scapular spine fracture* or scapular spine pathology* or scapular spine strain* or scapular spine stress*).mp.[mp=title, abstract, original title, name of substance word, subject heading word, floating sub-heading word, keyword heading word, organism supplementary concept word, protocol supplementary concept word, rare disease supplementary concept word, unique identifier, synonyms]
Embase
1. ('reverse shoulder prosthesis'/exp OR 'reverse shoulder prosthesis' OR (reverse AND ('shoulder'/exp OR shoulder) AND ('prosthesis'/exp OR prosthesis)) OR ('reverse shoulder arthroplasty'/exp OR 'reverse shoulder arthroplasty' OR (reverse AND ('shoulder'/exp OR shoulder) AND ('arthroplasty'/exp OR arthroplasty)) OR RTSA OR ('reverse total shoulder arthroplasty'/exp OR 'reverse total shoulder

arthroplasty' OR (‘reverse AND ('total'/exp OR total) AND ('shoulder'/exp OR shoulder) AND ('arthroplasty'/exp OR arthroplasty)) OR ('reverse total shoulder prosthesis'/exp OR 'reverse total shoulder prosthesis' OR (reverse AND ('total'/exp OR total) AND ('shoulder'/exp OR shoulder) AND ('prosthesis'/exp OR prosthesis)))

2. ('acromial fracture'/exp OR 'acromial fracture' OR (acromial AND ('fracture'/exp OR fracture)) OR ('acromial pathology' OR (acromial AND ('pathology'/exp OR pathology)) OR ('acromial stress' OR (acromial AND ('stress'/exp OR stress)) OR ('acromial strain' OR (acromial AND ('strain'/exp OR strain)))
3. ('scapular spine stress' OR (scapular AND ('spine'/exp OR spine) AND ('stress'/exp OR stress)) OR ('arthroplasty'/exp OR arthroplasty))) AND ('scapular spine stress' OR (scapular AND ('spine'/exp OR spine) AND ('stress'/exp OR stress)) OR ('scapular spine pathology' OR (scapular AND ('spine'/exp OR spine) AND ('pathology'/exp OR pathology)) OR ('scapular spine fracture' OR (scapular AND ('spine'/exp OR spine) AND ('fracture'/exp OR fracture)))

Pubmed

1. ("reversal"[All Fields] OR "reversals"[All Fields] OR "reverse"[All Fields] OR "reversed"[All Fields] OR "reversely"[All Fields] OR "reverses"[All Fields] OR "reversibilities"[All Fields] OR "reversibility"[All Fields] OR "reversible"[All Fields] OR "reversing"[All Fields] OR "reversion"[All Fields] OR "reversions"[All Fields]) AND ("shoulder prosthesis"[MeSH Terms] OR ("shoulder"[All Fields] AND "prosthesis"[All Fields]) OR "shoulder prosthesis"[All Fields] OR ("arthroplasty"[MeSH Terms] OR "arthroplasty"[All Fields] OR "arthroplasties"[All Fields])
2. (“RTSA”)

3. ("reversal"[All Fields] OR "reversals"[All Fields] OR "reverse"[All Fields] OR "reversed"[All Fields] OR "reversely"[All Fields] OR "reverses"[All Fields] OR "reversibilities"[All Fields] OR "reversibility"[All Fields] OR "reversible"[All Fields] OR "reversing"[All Fields] OR "reversion"[All Fields] OR "reversions"[All Fields]) AND ("total"[All Fields] OR "totaled"[All Fields] OR "totaling"[All Fields] OR "totalled"[All Fields] OR "totalling"[All Fields] OR "totals"[All Fields]) AND ("shoulder prosthesis"[MeSH Terms] OR ("shoulder"[All Fields] AND "prosthesis"[All Fields]) OR "shoulder prosthesis"[All Fields] OR ("reversal"[All Fields] OR "reversals"[All Fields] OR "reverse"[All Fields] OR "reversed"[All Fields] OR "reversely"[All Fields] OR "reverses"[All Fields] OR "reversibilities"[All Fields] OR "reversibility"[All Fields] OR "reversible"[All Fields] OR "reversing"[All Fields] OR "reversion"[All Fields] OR "reversions"[All Fields]) AND ("arthroplasty, replacement, shoulder"[MeSH Terms] OR ("arthroplasty"[All Fields] AND "replacement"[All Fields] AND "shoulder"[All Fields]) OR "shoulder replacement arthroplasty"[All Fields] OR ("total"[All Fields] AND "shoulder"[All Fields] AND "arthroplasty"[All Fields]) OR "total shoulder arthroplasty"[All Fields])
4. ("scapular"[All Fields] AND ("spine"[MeSH Terms] OR "spine"[All Fields] OR "spines"[All Fields] OR "spine s"[All Fields]) AND (("stress"[All Fields] OR "stressed"[All Fields] OR "stresses"[All Fields] OR "stressful"[All Fields] OR "stressfulness"[All Fields] OR "stressing"[All Fields]) OR ("pathology"[MeSH Terms] OR "pathology"[All Fields] OR "pathologies"[All Fields] OR "pathology"[MeSH Subheading]) OR ("scapular"[All Fields] AND ("spine"[MeSH Terms] OR "spine"[All Fields] OR "spines"[All Fields] OR "spine s"[All Fields]) AND ("sprains and strains"[MeSH Terms] OR ("sprains"[All Fields] AND "strains"[All Fields]) OR

"sprains and strains"[All Fields] OR "strain"[All Fields] OR "strains"[All Fields] OR "strain s"[All Fields]) OR ("scapular"[All Fields] AND ("spinal fractures"[MeSH Terms] OR ("spinal"[All Fields] AND "fractures"[All Fields]) OR "spinal fractures"[All Fields] OR ("spine"[All Fields] AND "fracture"[All Fields]) OR "spine fracture"[All Fields]))

5. ("acromial"[All Fields] AND ("stress"[All Fields] OR "stressed"[All Fields] OR "stresses"[All Fields] OR "stressful"[All Fields] OR "stressfulness"[All Fields] OR "stressing"[All Fields]) OR ("pathology"[MeSH Terms] OR "pathology"[All Fields] OR "pathologies"[All Fields] OR "pathology"[MeSH Subheading]) OR ("sprains and strains"[MeSH Terms] OR ("sprains"[All Fields] AND "strains"[All Fields]) OR "sprains and strains"[All Fields] OR "strain"[All Fields] OR "strains"[All Fields] OR "strain s"[All Fields]) OR ("fractur"[All Fields] OR "fractural"[All Fields] OR "fracture s"[All Fields] OR "fractures, bone"[MeSH Terms] OR ("fractures"[All Fields] AND "bone"[All Fields]) OR "bone fractures"[All Fields] OR "fracture"[All Fields] OR "fractured"[All Fields] OR "fractures"[All Fields] OR "fracturing"[All Fields]))

Cochrane

1. (RTSA [Title/Abstract/Keywords/MeSH Terms] OR reverse total shoulder arthroplasty[Title/Abstract/Keywords/MeSH Terms] OR reverse total shoulder prosthesis[Title/Abstract/Keywords/MeSH Terms] OR reverse shoulder arthroplasty[Title/Abstract/Keywords/MeSH Terms] OR reverse shoulder prosthesis[Title/Abstract/Keywords/MeSH Terms]) AND (acromial fracture[Title/Abstract/Keywords/MeSH Terms]) OR acromial stress[Title/Abstract/Keywords/MeSH Terms]) OR acromial strain[Title/Abstract/Keywords/MeSH Terms]) OR acromial

pathology[Title/Abstract/Keywords/MeSH Terms]) OR scapular spine

fracture[Title/Abstract/Keywords/MeSH Terms]) OR scapular spine

stress[Title/Abstract/Keywords/MeSH Terms]) OR scapular spine

strain[Title/Abstract/Keywords/MeSH Terms]) OR scapular spine

pathology[Title/Abstract/Keywords/MeSH Terms]))
